# Mechanistic Insights into *Lactobacillus harbinensis* and Other Probiotics Regulating Lipid Metabolism in T2DM Mice via the PPARγ-LXRα-NPC1L1 Signaling Pathway Based on Multi-Omics Analysis

**DOI:** 10.3390/metabo16030157

**Published:** 2026-02-27

**Authors:** Baheban Yeerjiang, Tabusi Manaer, Xuelian Liu, Reziya Bieerdimulati, Xinhua Nabi

**Affiliations:** 1 School of Pharmacy, Xinjiang Medical University, Urumchi 830017, China; 2 Xinjiang Key Laboratory of Biopharmaceuticals and Medical Devices, Urumchi 830017, China; 3 Xinjiang Key Laboratory of Natural Medicines Active Components and Drug Release Technology, Urumchi 830017, China

**Keywords:** type 2 diabetes mellitus, composite probiotics derived from fermented camel milk (CPCM), PPAR signaling pathway, intestinal flora, lipid metabolism

## Abstract

**Background/Objectives**: Intestinal dysbiosis is a pivotal trigger of type 2 diabetes mellitus (T2DM). Our previous studies confirmed that composite probiotics derived from fermented camel milk (CPCM), containing *Lactobacillus harbinensis* and 13 other strains, can ameliorate glucose and lipid metabolism in T2DM mice by reshaping bile acid profiles, and its effect may be associated with the PPARγ-LXRα-NPC1L1 signaling pathway. **Methods**: Metagenomic analysis characterized alterations in intestinal microbiota structure and functional genes post-CPCM intervention, proteomic analysis detected changes in protein expression profiles related to glucose and lipid metabolism in mice, and Caco-2 cells were used for in vitro validation to clarify the regulatory effect of exopolysaccharides (EPS) (the active component of CPCM) on the PPARγ-LXRα-NPC1L1 signaling pathway. **Results**: The results showed that CPCM significantly improved glucose and lipid metabolism and remodeled the intestinal flora structure in mice, markedly enriching beneficial bacteria such as *Lactobacillus* and *Akkermansia* and enhancing the expression of functional genes related to the peroxisome proliferator-activated receptor (PPAR) signaling pathway and short-chain fatty acid synthesis in the microbiota. Proteomic analysis revealed that CPCM reversed the expression of key proteins involved in fatty acid oxidation and transport, thereby restoring the function of the PPAR signaling pathway. In vitro experiments validated that extracellular polysaccharides, the active component of CPCM, significantly upregulated the expression of PPARγ and liver X receptor α (LXRα) and inhibited the expression of Niemann–Pick C1-Like 1 (NPC1L1), a cholesterol absorption transporter, in Caco-2 cells. **Conclusions**: In conclusion, CPCM ameliorates glucose and lipid metabolic disorders in T2DM through multiple mechanisms: reshaping the intestinal probiotic community, enhancing its beneficial metabolic functions, restoring the activity of the PPARγ-LXRα signaling pathway, and subsequently downregulating NPC1L1.

## 1. Introduction

Diabetes mellitus (DM) is a major global public health challenge with a continuously rising incidence, and it has become one of the leading chronic metabolic diseases threatening human health [1]. According to the latest data from the International Diabetes Federation (IDF), approximately 589 million adults worldwide suffer from diabetes, and this number is projected to rise to 853 million by 2050 [2], among which more than 90% are cases of T2DM [3]. The intestinal flora participates in the development of T2DM through its metabolome, with 60–70% of cases associated with gut microbiota disruption caused by unhealthy diets. Lifestyle interventions such as dietary optimization and regular exercise can effectively improve blood glucose levels [4].

Intestinal dysbiosis in T2DM is mainly characterized by reduced species richness and diversity of the microbiota [5]. The structural alterations of the intestinal flora are primarily manifested as an increase in harmful bacteria: the proportions of opportunistic pathogens or detrimental bacteria, including *Ruminococcus*, *Fusobacterium*, *Escherichia coli*, and *Desulfovibrio*, are elevated [6,7]. Meanwhile, beneficial bacteria such as *Bifidobacterium*, *Bacteroides*, and butyrate-producing bacteria (e.g., *Roseburia* and *Eubacterium rectale*) exhibit decreased abundance in the intestines of T2DM patients [8,9]. Additionally, it is accompanied by impaired functional stability [10]. The overgrowth of harmful bacteria leads to intestinal barrier dysfunction, allowing endotoxins such as lipopolysaccharide (LPS) to enter the circulatory system, which activates inflammatory signaling pathways, induces insulin resistance, and accelerates the progression of diabetes [11,12]. Beneficial bacteria ferment dietary fiber to produce short-chain fatty acids (SCFAs) such as butyrate and propionate, which can enhance insulin sensitivity and regulate intestinal barrier function [13]. A reduction in SCFA-producing bacteria in diabetic patients results in decreased SCFA levels, thereby disrupting glucose metabolism [14]. The intestinal flora also participates in bile acid transformation; secondary bile acids can regulate blood glucose by activating farnesoid X receptor (FXR) and G protein-coupled bile acid receptor 5 (TGR5) [15].

Studies have indicated that the Kazakh population in Xinjiang, China, exhibits a relatively low prevalence of diabetes despite a dietary pattern characterized by high fat and low vegetable intake. Within this population, a mutation in the *LIMA1-K306fs* gene, which is involved in cholesterol absorption, has been identified in families presenting with low blood lipid levels [16]. Conversely, evidence suggests that urban Kazakh children in Xinjiang have a higher obesity rate compared to their counterparts in other urban groups [17]. As obesity is a crucial risk factor for metabolic diseases, could this phenomenon be driven by intestinal microecological changes? After these children move to urban areas, their intestinal flora structure undergoes significant alterations, with the original dominant flora gradually disappearing, which may contribute to the increased obesity rate. This observation raises a key question: could the probiotics contained in fermented dairy products, which are consumed regularly, be associated with the *LIMA1* gene mutation?

Our research team previously confirmed that traditional fermented camel milk from Xinjiang exerts anti-diabetic effects. Fourteen strains of milk-derived probiotics with excellent probiotic properties (acid resistance, bile salt resistance, and strong adhesion) were isolated, and the CPCM was formulated through an optimized combination [18,19,20,21]. CPCM has demonstrated outstanding hypoglycemic and lipid-regulating effects in two distinct diabetic models. Further investigation into its mechanism of action revealed that CPCM can repair the intestinal barrier, effectively alleviate low-grade inflammation by inhibiting the Toll-like receptor (TLR)/myeloid differentiation factor 88 (MyD88)/nuclear factor kappa-B (NF-κB) pathway, and regulate macrophage polarization [19]. CPCM can increase the levels of intestinal SCFAs, elevate the contents of propionate and butyrate, and promote the secretion of glucagon-like peptide-1 (GLP-1) and peptide YY (PYY) by activating G protein-coupled receptor 43/41 (GPR43/41), thereby regulating blood lipid and glucose levels [22]. Regarding the significant lipid-lowering effects of CPCM, in vitro experiments have shown that while all 14 single strains exhibit good cholesterol-lowering activity, the efficacy of CPCM is significantly superior to that of any of these individual strains [23]. We designed experiments to investigate the effects of CPCM on bile acid metabolism; the results showed that serum total bile acid levels decreased while fecal total bile acid levels increased. Meanwhile, the proportions of endogenous agonists of FXR and TGR5 (lithocholic acid (LCA) and chenodeoxycholic acid (CDCA)) in bile acids increased, whereas FXR inhibitors (tauro-α-muricholic acid (T-α-MCA) and tauro-β-muricholic acid (T-β-MCA)) decreased. These changes synergistically promoted the expression of FXR and TGR5 in the liver, further upregulated the expression of PPARγ and LXRα in the colon, and significantly downregulated the expression of NPC1L1, a key protein responsible for intestinal cholesterol absorption [24]. Collectively, these findings suggest that CPCM may inhibit NPC1L1-mediated intestinal cholesterol absorption by activating the PPARγ-LXRα signaling axis, thereby regulating lipid metabolism.

Numerous studies have confirmed that industrialization, the widespread use of drugs (especially antibiotics), and changes in lifestyle have profound impacts on the composition and function of the intestinal flora [25]. These factors can induce imbalances in the host intestinal flora structure, which in turn disrupt metabolic homeostasis by causing glucose and lipid metabolism disorders. Probiotics have demonstrated distinct advantages in the prevention and treatment of T2DM [26,27]. Our team’s previous research has identified the multiple roles of CPCM in regulating glucose and lipid metabolism, but the underlying mechanisms by which CPCM modulates metabolism through the PPARγ-LXRα-NPC1L1 signaling pathway, particularly how the intestinal flora mediates the activation of this pathway, remain insufficiently elucidated. Therefore, this study systematically reveals the mechanism of action of composite probiotics in ameliorating T2DM-related metabolic disorders through animal model experiments, proteomic and metagenomic analyses, and in vitro validation for in-depth molecular mechanism research.

## 2. Materials and Methods

### 2.1. Source and Culture of Strains

In this study, ten lactic acid bacteria and four yeasts were isolated from traditional fermented camel milk sourced from the Xinjiang region of China, as identified by the Institute of Microbiology, Chinese Academy of Agricultural Sciences. Based on the current taxonomy of lactic acid bacteria (LAB) (http://lactobacillus.ualberta.ca/, accessed on 1 October 2025) [19], the identified LAB included strains such as *Lactobacillus harbinensis*, *Lactobacillus helveticus*, and *Lacticaseibacillus paracasei subsp. tolerans*, among others. The yeast species included *Issatchenkia orientalis* and *Kluyveromyces marxianus*, among others. Ten *Lactobacillus* strains and four yeast strains were, respectively, cultured in Man Rogosa Sharpe Broth and modified Man Rogosa Sharpe Broth at 37 °C for 48 h. The bacteria were collected by centrifugation (Heraeus Fresco 17× *g*, Thermo Fisher Scientific, Inc., Waltham, MA, USA) at 8000 rpm for 10 min, washed twice with phosphate-buffered saline (PBS), and suspended in physiological saline. The low-dose CPCM was prepared at a concentration of 1 × 10^8^ CFU/mL for the ten *Lactobacillus* strains and 1 × 10^6^ CFU/mL for the four yeast strains. The high-dose CPCM was prepared at a concentration of 1 × 10^10^ CFU/mL for the ten *Lactobacillus* strains and 1 × 10^8^ CFU/mL for the four yeast strains.

### 2.2. Animals and Treatments

Specific pathogen-free (SPF) male *db/db* mice and *db/m* mice (6 weeks old) were obtained from Changzhou Cavins Laboratory Animal Co., Ltd (Changzhou, Jiangsu, China). All animals were housed under controlled conditions (temperature: 22 ± 1 °C, humidity: 50 ± 5%, 12-h light/dark cycle) with free access to food and water. The experimental procedures were approved by the Institutional Animal Ethics Committee (Approval No. IACUC-20220312-10). Thirty-two *db/db* mice were randomly assigned to four groups (n = 8 per group): model group, positive control (metformin) group, low-dose CPCM group, and high-dose CPCM group. An additional eight *db/m* mice served as the normal control group. The control and model groups received physiological saline (0.4 mL/day) by gavage, while the metformin group was administered metformin (0.3 g/kg/day). The experimental groups received either low- or high-dose CPCM via daily gavage. After 8 weeks of intervention, the mice were fasted for 8 h and anesthetized via intraperitoneal injection of ketamine and diazepam. Blood, fecal, and liver tissue samples were then collected. The sample sizes used for each statistical analysis are indicated in the corresponding figures or figure legends.

### 2.3. Biochemical Parameter Analysis

Blood samples were obtained from the tail vein of the mice at weekly intervals from week 0 to week 8. Fasting blood glucose (FBG) was measured weekly throughout the 8-week period using a glucometer (HMD Biomedical, Taiwan, China). At the end of the 8th week, an oral glucose tolerance test (OGTT) was performed in the morning with a glucose dose of 2 g/kg. The area under the curve (AUC) for glucose was calculated based on measurements taken at different time points. The levels of HbA1c and C-peptide were determined using enzyme-linked immunosorbent assay (ELISA) kits (Elabscience Biotechnology Co., Ltd., Wuhan, China). Serum levels of total cholesterol (TC), triglycerides (TG), low-density lipoprotein cholesterol (LDL-C), and high-density lipoprotein cholesterol (HDL-C) were measured using a fully automated biochemical analyzer.

### 2.4. Metagenomic Sequencing

Fecal samples were collected and stored at –80 °C until processing. Genomic DNA was extracted using the FastPure Stool DNA Isolation Kit (Magnetic bead) (MJYH, Shanghai, China). The concentration and purity of the DNA were assessed, and its integrity was verified using 1% agarose gel electrophoresis. The DNA was fragmented to an average size of approximately 350 bp using a Covaris M220 focused-ultrasonicator (Covaris, LLC, Woburn, MA, USA; distributed by Gene Company Limited, Shanghai, China). A paired-end sequencing library was then constructed with the NEXTFLEX Rapid DNA-Seq Kit (Bioo Scientific, Austin, TX, USA) and subjected to metagenomic sequencing on the Illumina NovaSeq™ X Plus platform (Illumina, San Diego, CA, USA) at Shanghai Meiji Biomedical Technology Co., Ltd. (Shanghai, China).

#### 2.4.1. Processing of Metagenome Sequencing Data

The data were analyzed on the free online platform of Majorbio Cloud Platform (www.majorbio.com, accessed on 4 October 2025). Briefly, the raw sequencing reads were trimmed of adapters, and low-quality reads (length < 50 bp or with average quality value < 20) were removed by fastp (https://github.com/OpenGene/fastp, accessed on 4 October 2025, version 0.20.0). Reads were aligned to the host genome by BWA (http://bio-bwa.sourceforge.net, accessed on 4 October 2025, version 0.7.17), and any hits associated with the reads and their paired reads were removed. High-quality reads were assembled de novo using MEGAHIT [28] (v1.1.2). Open reading frames were predicted from the assembled contigs using Prodigal (v2.6.3), and non-redundant gene catalogs were constructed with CD-HIT (v4.7).

#### 2.4.2. Taxonomic and Functional Annotation

The best-hit taxonomy of non-redundant genes was obtained by aligning them against the NCBI NR database by DIAMOND (http://ab.inf.uni-tuebingen.de/software/diamond/, accessed on 4 October 2025, version 2.0.13) with an e-value cutoff of 1 × 10^−5^. Similarly, the functional annotation (NR, KEGG) of non-redundant genes was obtained. Based on the taxonomic and functional annotation and the abundance profile of non-redundant genes, the differential analysis was carried out at each taxonomic, functional, or gene-wise level by the Kruskal–Wallis test.

### 2.5. Proteomics Analysis

All experiments were conducted in collaboration with and under the guidance of Majorbio [29]. Liver tissue samples were processed while frozen. Each sample was placed into an MP shock tube (MP Biomedicals, LLC, Irvine, CA, USA) containing an appropriate volume of protein lysis buffer and homogenized three times for 180 s each using a high-throughput tissue grinder (FastPrep-96™, MP Biomedicals, LLC, Irvine, CA, USA). Subsequently, the samples underwent non-contact low-temperature ultrasonication for 30 min, followed by centrifugation at 8 °C and 14,000× *g* for 15 min. The supernatant was collected, and protein concentration was determined using a BCA assay kit (Thermo Fisher Scientific, Inc., Waltham, MA, USA) according to the manufacturer’s protocol.

A total of 100 μg of protein from each sample was redissolved in 100 mM triethylammonium bicarbonate (TEAB) buffer, reduced with 10 mM Tris (2-carboxyethyl) phosphine (TCEP) at 37 °C for 60 min, and alkylated with 40 mM iodoacetamide (IAM) at room temperature in the dark for 40 min. After centrifugation at 10,000× *g* and 4 °C for 20 min, the pellet was collected and resuspended in 100 μL of 100 mM TEAB buffer. Trypsin was added at a 1:50 (enzyme-to-protein) ratio, and the mixture was incubated at 37 °C overnight.

Following tryptic digestion, peptides were vacuum-dried, reconstituted in 0.1% trifluoroacetic acid (TFA), and desalted using HLB solid-phase extraction columns before being dried again in a vacuum concentrator (Centrifuge 5424 R, Eppendorf AG, Hamburg, Germany). Peptide quantification was performed via UV absorbance using a NANO DROP ONE instrument (Thermo Fisher Scientific, Inc., Waltham, MA, USA). Based on the quantification results, the peptides were analyzed on a Vanquish Neo UHPLC system coupled to an Orbitrap Astral mass spectrometer (Thermo Fisher Scientific, Inc., Waltham, MA, USA) at Majorbio Bio-Pharm Technology Co., Ltd. (Shanghai, China). Briefly, separation was carried out on a uPAC High-Throughput column (75 μm × 5.5 cm, Thermo) with mobile phases consisting of solvent A (2% acetonitrile, 0.1% formic acid in water) and solvent B (80% acetonitrile, 0.1% formic acid in water) over an 8 min chromatographic gradient. Data-independent acquisition (DIA) was performed on this instrument in DIA mode, with a mass scan range of 100–1700 *m*/*z*.

### 2.6. Cell Experiments

#### 2.6.1. Extraction of Exopolysaccharides

Activated bacterial strains were cultured, and the culture broth containing probiotic EPS was centrifuged (4 °C, 10,000 rpm, 10 min) to collect the supernatant. The supernatant was concentrated to one-fifth of its original volume using a rotary evaporator (Scientific Industries, Inc., Bohemia, NY, USA). Trichloroacetic acid solution was added to the concentrate to a final concentration of 4% (*w*/*v*), followed by magnetic stirring at room temperature for 30 min. The mixture was then centrifuged (4 °C, 10,000 rpm, 15 min), and the resulting supernatant was collected. To precipitate the EPS, 2–3 volumes of anhydrous ethanol were added to the supernatant, and the solution was kept at 4 °C for 24 h. After precipitation, the mixture was centrifuged (4 °C, 1000× *g*, 20 min). The supernatant was discarded, and the EPS pellet was collected, dissolved in an appropriate volume of ultrapure water, and transferred to dialysis bags (molecular weight cutoff: 8–14 kDa). Dialysis was performed with ultrapure water at 4 °C for 3 days, with water changes every 4 h (including overnight). Following dialysis, the EPS solution was poured into culture dishes and freeze-dried for 24 h to obtain crude EPS.

#### 2.6.2. Impact of EPS on the Viability of Caco-2 Cells

Caco-2 cells were harvested at full confluence, followed by digestion and centrifugation at 1000 rpm for 5 min. The cell pellet was resuspended and counted, then seeded into 96-well plates at a density of 6000 cells per well. Each experimental group received 100 μL of treatment medium containing varying concentrations of cholesterol micelles and EPS suspension. To minimize edge effects, the outermost wells of the plate were filled with 150 μL of sterile PBS buffer.

After a 24 h incubation under standard culture conditions, 10 μL of MTT reagent was added to each well in the dark. The plates were further incubated for 4 h, after which the culture medium was carefully aspirated. Subsequently, 150 μL of DMSO was added to each well to solubilize the formazan crystals. The plates were gently shaken and allowed to stand until complete dissolution. Absorbance was measured at 570 nm, and the cell survival rate was calculated using the following formula:

Cell survival rate (%) = (OD values for each sample group/Blank group OD value) × 100%

#### 2.6.3. Establishment of the Cholesterol Absorption Model in Caco-2 Cells

Confluent Caco-2 cell monolayers were used for the experiment. The complete culture medium was aspirated, and the cells were washed twice with phosphate-buffered saline (PBS). Subsequently, the cells were equilibrated in serum-free Dulbecco’s Modified Eagle Medium (DMEM) for 1 h.

Following equilibration, the cells were allocated into four experimental groups: a normal control group, a cholesterol absorption model group, and low-, medium-, and high-dose EPS treatment groups. The normal control group was incubated with serum-free DMEM only. To establish the cholesterol absorption model, the model group was treated with a cholesterol-mixed micelle solution. This solution contained 0.2 mM cholesterol, 2 mM sodium taurocholate, and 0.2% (*w*/*v*) fatty acid-free bovine serum albumin (BSA) in serum-free DMEM.

#### 2.6.4. The Effect of EPS on Cholesterol Uptake in Caco-2 Cells

Cells were divided into the following groups: normal control, cholesterol absorption model, and low-, medium-, and high-dose EPS intervention groups. The normal control group received basal medium only. The model group was treated with a cholesterol micelle solution to establish the cholesterol absorption model. Each intervention group was co-treated with the cholesterol micelle solution and EPS at low (10 μg/mL), medium (40 μg/mL), or high (160 μg/mL) concentrations. The EPS solution was prepared by dissolving the EPS in serum-free DMEM, followed by filter-sterilization through a 0.22 μm membrane and dilution to the required concentrations. Cells were then incubated for 24 h, after which the cellular cholesterol content was measured. After the treatment period, the medium in each well was aspirated and discarded. The cells were washed with sterile PBS, detached using trypsin, and collected by centrifugation at 1000 rpm for 5 min at 4 °C. The supernatant was discarded, and the cell pellet was retained for subsequent analysis. TC levels were quantified using a commercial assay kit (Jiancheng Bioengineering Institute, Nanjing, China).

#### 2.6.5. Quantitative Real-Time PCR

RNA was isolated from colon tissues using Trizol reagent (TAKARA, TransGEN Biotech Co., Beijing, China). The total RNA was used to synthesize cDNA with a First Strand cDNA Synthesis Kit (Servicebio, Wuhan, China). The amplification and mRNA levels were determined by quantitative real-time PCR. The relative mRNA expression was calculated using the 2^−ΔΔCt^ method. The primer sequences of the target genes are listed in Supplementary Table S1.

### 2.7. Data Analysis

Data are presented as mean ± standard deviation (mean ± SD). Differences between groups were compared using one-way ANOVA. Significant differences were indicated as *p* < 0.05, *p* < 0.01, and *p* < 0.001. All statistical analyses were performed using SPSS 27.0. All statistical charts were plotted using GraphPad Prism software (Version 8.0.1, GraphPad Software, LLC, San Diego, CA, USA). Schematic diagrams of molecular mechanisms were designed with BioRender (BioRender.com, accessed on 4 October 2025). Metagenomic sequencing of fecal samples and proteomic analysis of liver specimens were both performed by Shanghai Meiji Biotechnology Co., Ltd. Raw sequencing data underwent bioinformatics processing through the company’s established workflow, with resulting figures downloaded from the company’s official website (URL: www.majorbio.com, accessed on 4 October 2025).

For metagenomic KEGG pathway enrichment analysis and proteomic differential expression analysis, the Benjamini–Hochberg (BH) method was applied for multiple testing correction to control the false discovery rate (FDR), and the adjusted *p*-values were reported as “*p adjust*”.

#### Statistical Analyses of Proteomics

Bioinformatic analysis of proteomic data was performed with the Majorbio Cloud platform (https://cloud.majorbio.com, accessed on 29 September 2025). *p*-values and fold change (FC) for the proteins between the two groups were calculated using the R package’s “*t*-test” (R version 4.2.1). The thresholds of fold change (>1.2) and *p*-value < 0.05 were used to identify differentially expressed proteins (DEPs). Functional annotation of all identified proteins was performed using GO (http://geneontology.org/, accessed on 29 September 2025) and KEGG pathway (http://www.genome.jp/kegg/, accessed on 29 September 2025). DEPs were further used for GO and KEGG enrichment analysis. Protein–protein interaction analysis was performed using String v11.5.

## 3. Results

### 3.1. Effect of CPCM on Glucose and Lipid Metabolism in db/db Mice

#### 3.1.1. Effect of CPCM on Glycated Hemoglobin (HbA1c) and C-Peptide (CP) in *db/db* Mice

**Figure 1:**
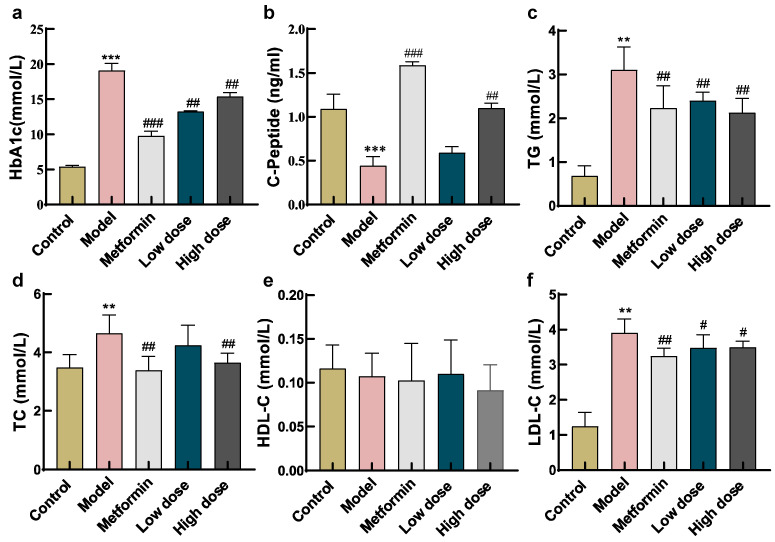
Impact of CPCM on serum biochemical biomarkers in *db/db* mice. Serum levels of (**a**) HbA1c and (**b**) C-peptide were determined by ELISA kits (n = 3); (**c**) TG, (**d**) TC, (**e**) HDL-C, and (**f**) LDL-C were measured (n = 8). Data are presented as mean ± SD values. ** *p* < 0.01, *** *p* < 0.001 vs. control group; ^#^ *p* < 0.05, ^##^ *p* < 0.01, ^###^ *p* < 0.001 vs. model group.

HbA1c levels were significantly higher in the model group than in the control group (*p* < 0.01). After eight weeks of treatment, both the low- and high-dose CPCM groups exhibited significant reductions in HbA1c compared to the model group (*p* < 0.001), with the metformin group showing the most notable decrease. Furthermore, the high-dose CPCM group achieved a significantly greater reduction in HbA1c compared to the low-dose group (*p* < 0.01) (Figure 1a).

CP levels were also measured across all groups. Following the 8-week intervention, the model group displayed the lowest CP values, consistent with the diabetic phenotype. Compared to the model group, CP levels were significantly elevated in both the metformin group (*p* < 0.001) and the high-dose CPCM group (*p* < 0.01). Although the low-dose CPCM group demonstrated an upward trend in CP levels, the change was not statistically significant (*p* > 0.05) (Figure 1b).

#### 3.1.2. Effect of CPCM on Serum Lipid Profiles (TC, TG, LDL-C, and HDL-C) in *db/db* Mice

Compared to the control group, mice in the model group exhibited significantly elevated serum levels of TG (Figure 1c), TC (Figure 1d), and LDL-C (Figure 1f) (*p* < 0.01). In contrast, both the high- and low-dose CPCM groups demonstrated significant reductions in TG and LDL-C relative to the model group (*p* < 0.05). Furthermore, the high-dose group showed a marked decrease in TC (*p* < 0.01). However, no statistically significant differences in HDL-C were observed among the groups (Figure 1e).

#### 3.1.3. Effect of CPCM on Fasting Blood Glucose Levels in *db/db* Mice

Given that impaired glucose tolerance is a hallmark of T2DM, FBG levels were monitored at two-week intervals throughout the study. Prior to the intervention, FBG levels in the model group were significantly higher than those in the control group (*p* < 0.001), while no significant differences were noted among the experimental groups (*p* > 0.05), confirming the successful establishment of the T2DM model and the validity of the randomized group allocation.

Throughout the experimental period, FBG levels remained consistently elevated in the model group compared to the control group (*p* < 0.001). Relative to the model group, significant reductions in FBG were observed in the metformin group from week 4 onwards (*p* < 0.05). Similarly, the low-dose group demonstrated a statistically significant decrease by week 6 (*p* < 0.01), while the high-dose group also exhibited a significant reduction at week 6 (*p* < 0.05), which became more pronounced by week 8 (*p* < 0.01). All results are summarized in Table 1.

#### 3.1.4. Effect of CPCM on Oral Glucose Tolerance Test (OGTT) in *db/db* Mice

The OGTT was conducted to assess glucose metabolism capacity in the experimental groups. At baseline, both OGTT values and the corresponding AUC were significantly elevated in the model group compared to the control group (*p* < 0.001). After the 8-week intervention, significant reductions in OGTT values and AUC were observed in the metformin, low-dose, and high-dose groups relative to the model group (*p* < 0.001). The metformin group demonstrated the most pronounced improvement, while the high-dose group exhibited a more robust glucose-lowering trend compared to the low-dose group. Detailed results are provided in Table 2.

#### 3.1.5. Effect of CPCM on Body Weight in *db/db* Mice

Prior to the intervention, the body weights in the model group were significantly higher than those in the control group (*p* < 0.001). Mice were subsequently randomly assigned to various treatment groups, with no significant differences in body weight observed among these groups at the study’s commencement (*p* > 0.05). During the first six weeks, body weight in the model group continued to increase, followed by a decline beginning in the seventh week. In comparison to the model group, significant reductions in body weight were noted from the sixth week onward in both the metformin group (57.53 ± 3.00 g) and the low-dose CPCM group (58.18 ± 3.00 g) (*p* < 0.05), with significant reductions also observed by the eighth week in the high-dose CPCM group (56.29 ± 3.48 g) (*p* < 0.05). Data are summarized in Supplementary Table S2.

### 3.2. Restructuring of Gut Microbiota Functional Genes by CPCM

#### 3.2.1. Effect of CPCM on Gut Microbiota Diversity in *db/db* Mice

**Figure 2:**
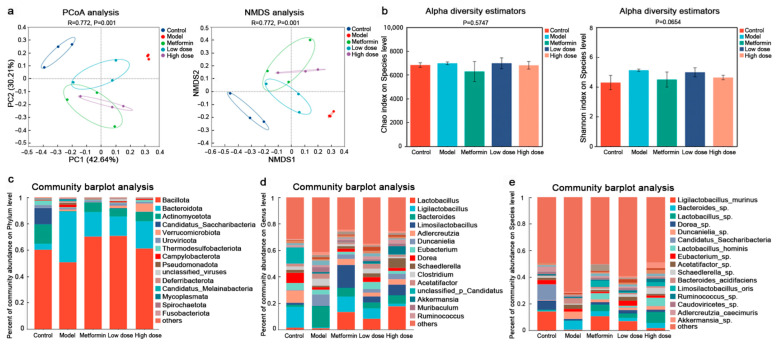
Metagenomic analysis of the impact of CPCM on gut microbiota diversity and community structure in *db/db* mice: (**a**) Multivariate statistical analyses (PCoA and NMDS) of gut microbiota *beta* diversity from control and *db/db* mice with or without CPCM intervention. (**b**) Alpha diversity analyses of gut microbiota in control and *db/db* mice with or without CPCM intervention. (**c**–**e**) Community bar-plot analyses of gut microbiota at the phylum, genus, and species levels, illustrating changes in community structure following CPCM intervention.

Metagenomic sequencing was conducted to characterize the gut microbial community structure across the mouse groups. The relative abundance of identified microbial taxa is detailed in Supplementary Table S3. Principal coordinate analysis (PCoA) and non-metric multidimensional scaling (NMDS) were employed to compare microbiome profiles among the five experimental groups (Figure 2a). In the PCoA, the first two principal coordinates accounted for 42.64% (PC1) and 30.21% (PC2) of the total variance, respectively. Both PCoA and NMDS revealed statistically significant separation (*p* < 0.01) among the groups, indicating considerable divergence in gut microbiota composition. Although overall α-diversity did not differ significantly across groups, the low-dose CPCM group exhibited a higher microbial abundance than the model group, as indicated by the Chao index (Figure 2b).

#### 3.2.2. CPCM Modulates the Gut Microbiota Structure in *db/db* Mice

At the phylum level, community composition analysis (Figure 2c) revealed distinct structural differences among the groups. *Bacillota* was the most abundant phylum across all groups. A notable disruption in the *Bacillota*/*Bacteroidota* ratio was observed, with a decrease in the model group and an increase in all other groups. The relative abundance of *Actinomycetota* was elevated in the metformin, low-dose CPCM, and high-dose CPCM groups.

At the genus level (Figure 2d), the model group exhibited higher proportions of *Bacteroides* and *Duncaniella*, both of which were reduced in the CPCM-treated groups. In contrast, *Lactobacillus* emerged as the dominant genus in the metformin, low-dose, and high-dose CPCM groups compared to the model group. Similarly, the abundance of *Ligilactobacillus* increased in these three intervention groups, mirroring its dominance in the control group. The abundances of *Limosilactobacillus* and *Adlercreutzia* were also elevated in the metformin and CPCM intervention groups, with the most pronounced increase observed in the metformin group. Additionally, a higher abundance of *Akkermansia* was detected in both the low-dose and high-dose CPCM groups.

At the species level (Figure 2e), *Ligilactobacillus murinus* was the dominant species in the control group and remained dominant in the metformin, low-dose, and high-dose CPCM groups. Certain unidentified species of *Dorea* and *Bacteroides* also constituted considerable proportions. In the model group, *Bacteroides* sp. was the dominant species, with abundance trends across groups aligning with those observed at the genus level. The metformin group was primarily composed of *Ligilactobacillus murinus*, while *Lactobacillus* sp. and *Bacteroides* sp. also demonstrated relatively high abundances. Compared to the model group, the abundance of *Bacteroides* sp. was significantly reduced in the CPCM-treated groups.

**Figure 3:**
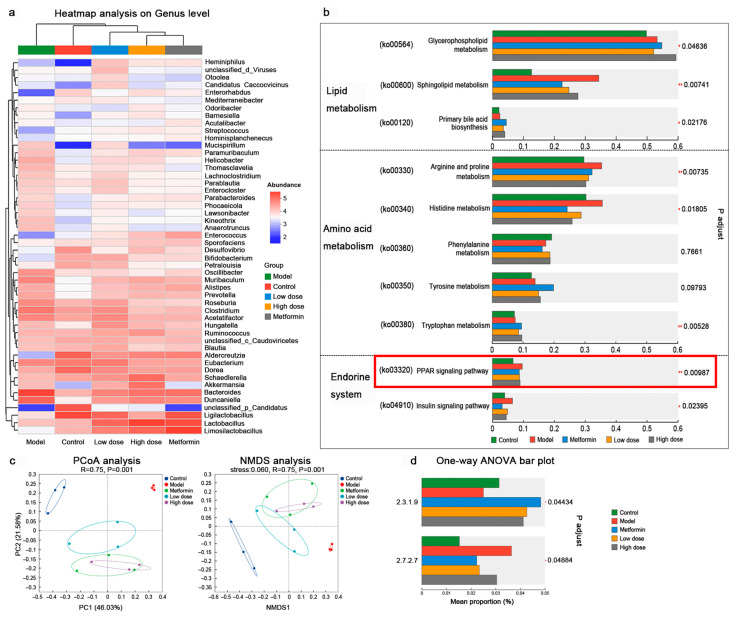
Metagenomic analysis of the impact of CPCM on gut microbiota composition and metabolic function in *db/db* mice: (**a**) Heatmap of the top 50 most abundant bacterial phyla in control and *db/db* mice with or without CPCM intervention. (**b**) KEGG pathway analysis comparing the metabolic functions of gut microbiota between control and *db/db* mice following CPCM intervention. The red box highlights the significantly enriched signaling pathway of interest. (**c**) Multivariate statistical analyses (PCA, PCoA, and NMDS) of the PPAR signaling pathway (KEGG ko03320) in the intestinal microbiota. (**d**) Changes in the relative abundance of acetyl-CoA transferase and butyrate kinase associated with CPCM intervention. * *p adjust* < 0.05; ** *p adjust* < 0.01.

Furthermore, the heatmap (Figure 3a) illustrates the abundance distribution of the top 50 microbial genera across the five experimental groups. The color intensity in each cell reflects the relative abundance of a specific genus within the corresponding group, with clear gradations distinguishing the groups. Pronounced variations were observed in key genera, including *Lactobacillus*, *Bacteroides*, and *Akkermansia*. Overall, the heatmap highlights marked differences in microbial abundance between CPCM-treated and model groups, collectively underscoring the substantial remodeling of the gut microbiota under T2DM conditions.

#### 3.2.3. Remodeling of the Gut Microbiota’s Functional Gene Profile by CPCM

KEGG pathway enrichment analysis revealed significant remodeling of the gut microbial functional gene profile following CPCM intervention compared to the model group. Multiple metabolic disease-related pathways were significantly enriched, including lipid metabolism, amino acid metabolism, and the endocrine system. Notably, within the endocrine system, the PPAR signaling pathway was prominently annotated in the CPCM-treated group (Figure 3b). Multivariate analyses, including PCoA and NMDS, further confirmed distinct bacterial distribution patterns associated with this pathway (Figure 3c), indicating that CPCM modulates the overall genetic capacity of the gut microbiota involved in PPAR signaling.

We next analyzed key microbial gene functions. Given that SCFAs, particularly butyrate, act as natural agonists of PPARγ, we concentrated on genes associated with SCFA synthesis. Acetyl-CoA transferase (EC 2.3.1.9), a pivotal enzyme in the synthesis of acetate and propionate, was significantly upregulated in the CPCM group compared to the model group (Figure 3d). An intriguing observation was made regarding butyrate kinase (EC 2.7.2.7), a critical enzyme for microbial butyrate production: its gene abundance was significantly elevated in the model group compared to the control, yet CPCM intervention led to a trend of reduction toward normal levels, although this was not statistically significant. We propose that T2DM impairs PPAR pathway activity, triggering a compensatory stress response in the gut microbiota, as evidenced by the upregulation of butyrate kinase. However, this compensatory mechanism is insufficient to counteract metabolic dysfunction. CPCM appears to directly restore PPAR pathway function while alleviating microbial stress, thereby synergistically improving glucose and lipid homeostasis.

The aforementioned fecal metagenomic analysis elucidated the regulatory effects of CPCM on the gut microbiota of mice with T2DM: on the one hand, probiotic intervention significantly remodelled the structure of the gut microbial community (with an increased abundance of bacteria that produce short-chain fatty acids, such as *Akkermansia*); on the other hand, the functional annotation of microbial genes revealed markedly different expression levels in modules related to lipid metabolism, such as primary bile acid biosynthesis.

As a key mediator of probiotic–host interactions, alterations in the structure and function of the gut microbiota can be transmitted to the liver via the bloodstream in the form of microbial metabolites. This regulates the activation of core hepatic lipid metabolism pathways [30]. To further elucidate the molecular bridge through which probiotics regulate the gut microbiota and thereby improve hepatic lipid metabolism, this study employed hepatic proteomics analysis. Focusing on proteins associated with the PPARγ-LXRα-NPC1L1 signaling pathway, we screened for differentially expressed proteins in the livers of T2DM mice following probiotic intervention. This investigation explores how gut microbiota remodeling improves lipid metabolism in *db/db* mice.

### 3.3. Effect of CPCM on Hepatic Protein Expression in db/db Mice

#### 3.3.1. Identification of Differentially Expressed Hepatic Proteins

**Figure 4:**
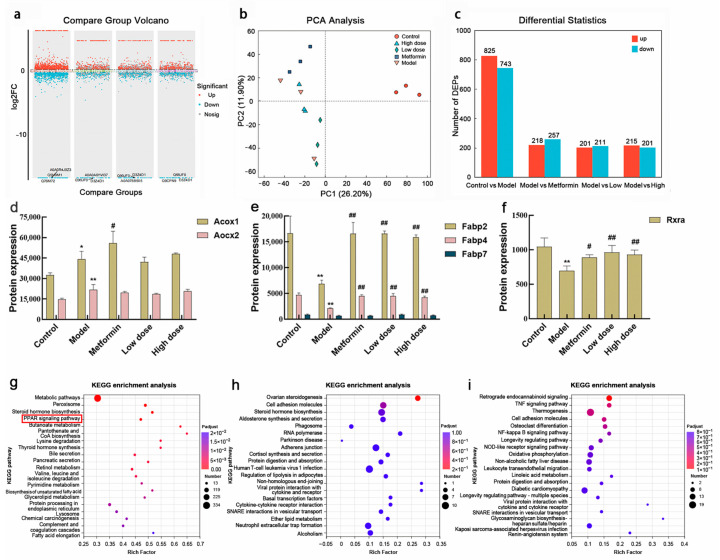
Proteomic analysis of the impact of CPCM on hepatic protein expression profiles in *db/db* mice (n = 3): (**a**) Volcano plot of differentially expressed proteins (DEPs) in the liver of control, model, and CPCM-treated *db/db* mice. (**b**) PCA of DEPs in the liver of the five experimental groups. (**c**) Comparative chart of DEPs among different comparison groups. (**d**–**f**) Protein expression levels of key hepatic proteins: (**d**) *Acox1* and *Acox2*, (**e**) *FABP1*, *FABP2*, *FABP4*, and *FABP7*, and (**f**) *Rxra*, in *db/db* mice following CPCM intervention (* *p* <0.05, ** *p* <0.01 vs. control; ^#^ *p* <0.05, ^##^ *p* <0.01 vs. model). (**g**–**i**) Comparative bubble charts of KEGG-enriched signaling pathways: (**g**) control vs. model group; (**h**) model vs. low-dose group; (**i**) model vs. high-dose group. Note: The horizontal axis represents the enrichment ratio, referring to the ratio of the number of proteins enriched in the pathway (protein number) to the number of proteins annotated to the pathway (background number). A higher ratio indicates a greater degree of enrichment; the vertical axis represents the KEGG pathway. Each bubble in the graph represents a KEGG pathway. The size of the bubble corresponds to the number of proteins enriched in the KEGG pathway. The different colors of the bubbles represent the *p*-value.

Using liquid chromatography–mass spectrometry (LC–MS) proteomic analysis, we investigated alterations in protein expression in the livers of *db/db* mice. Volcano plots illustrated the overall distribution of quantified proteins (Figure 4a). Principal component analysis (PCA) revealed similarities between the high-dose CPCM group and the metformin group, although some overlap with the model group was also noted. Conversely, the low-dose group exhibited a distinct separation from the model group (Figure 4b).

Differentially expressed proteins (DEPs) were identified across comparisons (Figure 4c). Among the 12,678 proteins quantified across the five experimental groups, 2871 exhibited significant variation. Compared to the model group, the low-dose group displayed 201 upregulated and 211 downregulated proteins, while the high-dose group showed 215 upregulated and 201 downregulated proteins (screening threshold: FC ≥ 1, *p* < 0.05). Supplementary Table S4 displays the top ten DEP.

Notably, among the DEPs in the low-dose group, several are associated with the pathogenesis of T2DM, including Atg13 (FC = 32), Cfd (FC = 0.00001), and Fabp3 (FC = 1.79). Importantly, the fatty acid-binding protein (FABP) family, to which Fabp3 belongs, serves as a key downstream regulator of the PPAR signaling pathway.

#### 3.3.2. Modulation of Key PPAR Signaling Pathway Proteins in the Liver by CPCM

Through functional annotation of the hepatic proteome, key proteins associated with the PPAR signaling pathway were identified. We first compared the expression profiles of these proteins between the control and model groups to confirm molecular alterations under T2DM conditions. As summarized in Table 3, the model group exhibited significant downregulation of multiple components of the PPAR pathway, including the following:

- Fatty acid *β*-oxidation rate-limiting enzymes (*ACOX1* and *ACOX2*), which indicate impaired hepatic fatty acid catabolism;
- Fatty acid transport proteins (*FABP1*, *FABP2*, *FABP4*, and *FABP7*), suggesting compromised uptake and intracellular trafficking of fatty acids;
- *RXRa*, the essential dimerization partner of PPARγ, whose reduction may attenuate PPARγ transcriptional activity.

To assess the therapeutic effect of CPCM, we further compared protein expression between the CPCM-treated and model groups. Notably, nearly all key PPAR pathway proteins—except ACOX—were significantly upregulated in the CPCM-treated group compared to the model group (*p* < 0.05). As illustrated in Figure 4d–f, following CPCM intervention, the expression levels of most PPAR-related proteins that were downregulated in the model group (including *ACOX1*, *ACOX2*, *FABP1*, *FABP2*, *FABP4*, *FABP7*, and *RXRa*) exhibited a consistent trend of reverting toward levels observed in the control group, indicating a restoration of pathway homeostasis.

#### 3.3.3. Effect of CPCM on Signaling Pathways of Differentially Expressed Proteins in the Liver of *db/db* Mice

KEGG pathway enrichment analysis of differentially expressed proteins was conducted to assess systemic alterations in the PPAR signaling pathway. A comparison between the model and control groups (Figure 4g) revealed significant enrichment of the PPAR signaling pathway (*p-adjust* < 0.05), indicating a substantial disruption of hepatic PPAR signaling under T2DM conditions. In contrast, while the PPAR pathway was successfully annotated in both the high- and low-dose CPCM groups relative to the model group, the enrichment did not achieve statistical significance. This finding corresponds with the restoration trend of individual protein expression described in Section 3.3.2, collectively demonstrating that CPCM intervention does not merely activate the PPAR pathway but rather normalizes the expression of dysregulated proteins within the pathway, thereby restoring the functional state of the entire PPAR signaling network from a diabetes-induced disorder to a relatively stable physiological level.

Further KEGG enrichment analyses conducted separately for the low- and high-dose groups revealed distinct intervention patterns between the two dosages. Following low-dose CPCM treatment, the hepatic proteome showed enrichment trends in pathways related to endocrine regulation and fundamental cellular functions (Figure 4h), including regulation of lipolysis in adipocytes, cell adhesion molecules, RNA polymerase, steroid hormone biosynthesis, aldosterone synthesis and secretion, and cortisol synthesis and secretion. These findings suggest that the low-dose intervention primarily targets systemic hormonal rebalancing and basic metabolic regulation.

In contrast, the high-dose group displayed enrichment trends in core pathways closely associated with inflammation, immunity, and energy metabolism (Figure 4i). Key enriched pathways included the NF-kappa B signaling pathway, oxidative phosphorylation, the NOD-like receptor signaling pathway, thermogenesis, retrograde endocannabinoid signaling, the TNF signaling pathway, and the renin–angiotensin system. These results indicate that high-dose CPCM exerts potent anti-inflammatory and immunomodulatory effects in the liver, with broad therapeutic implications across multiple domains, including inflammation, metabolism, immunity, and cardiovascular function relevant to T2DM pathogenesis and progression.

This study elucidated the hepatic mechanisms through which CPCM improves glucose and lipid metabolism in T2DM mice using liver proteomics. We first confirmed that the T2DM pathological state significantly disrupts the hepatic PPAR signaling pathway, as evidenced by the downregulation of core proteins including *ACOX1* and *ACOX2* (key enzymes in fatty acid *β*-oxidation), members of the fatty acid-binding protein (FABP) family, and *RXRa* (the essential heterodimerization partner of PPARγ). These alterations provide a molecular explanation for the hepatic lipid metabolic disorders observed in diabetes. Following CPCM intervention, a clear restorative effect towards metabolic homeostasis was observed. Although not all proteins reached statistical significance compared to the model group, their expression levels consistently exhibited a normalization trend. These findings demonstrate that CPCM facilitates the recovery of hepatic metabolic homeostasis by restoring the functional integrity of the PPAR signaling pathway.

In summary, our in vivo experiments demonstrate that CPCM operates through a dual-level mechanism: metagenomic analysis indicated that CPCM remodels gut microbiota function, such as by modulating SCFA synthesis genes, supporting its role in the gut–liver axis-mediated regulation of host metabolism. Meanwhile, hepatic proteomics revealed that CPCM restores homeostasis of the PPAR signaling pathway, reversing the T2DM-induced downregulation of key proteins (e.g., *ACOX*, *FABPs*, *RXRα*) and reestablishing near-normal pathway function.

However, this “microbiota–liver” model does not directly confirm whether CPCM regulates the PPARγ-LXRα-NPC1L1 signaling pathway. A fundamental question remains: Can CPCM or its active components directly interact with host intestinal cells and regulate this core pathway independently of the microbiota? To address this and provide direct validation of PPARγ-LXRα-NPC1L1 signaling, we designed an in vitro experiment. We extracted key active polysaccharides from CPCM and established a cholesterol absorption model using Caco-2 cells to investigate their effects.

### 3.4. Effect of CPCM-Derived Exopolysaccharides (EPS) on Cholesterol Absorption in Caco-2 Cells

#### 3.4.1. Effect of EPS and Cholesterol Micelles on the Viability of Caco-2 Cells

**Figure 5:**
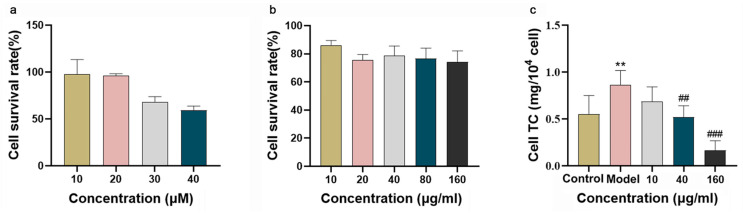
Impact of cholesterol micelles and EPS on Caco-2 cell viability and cholesterol uptake: (**a**) Effect of cholesterol micelle concentration on Caco-2 cell viability. (**b**) Effect of EPS concentration on Caco-2 cell viability. (**c**) Effect of EPS on cholesterol uptake in Caco-2 cells. Data are presented as mean ± SD (n = 6). ** *p* < 0.01 vs. control group; ^##^ *p* < 0.01, ^###^ *p* < 0.001 vs. model group.

To exclude potential cytotoxicity as a confounding factor, the effects of EPS and cholesterol micelles on Caco-2 cell viability were evaluated using the MTT assay. As illustrated in Figure 5a, treatment with cholesterol micelles at concentrations of 30 μM and 40 μM reduced cell viability to approximately 70%. In contrast, at 20 μM, cell viability remained above 95%, with cells maintaining normal morphology under microscopic observation. Similarly, as shown in Figure 5b, EPS treatment across a concentration range of 10–160 μg/mL resulted in cell viability exceeding 70%, with no observable adverse effects on cell growth. Based on these results, EPS concentrations of 10, 40, and 160 μg/mL were selected as the low-, medium-, and high-dose treatment groups, respectively, while cholesterol micelles at 20 μM were used for all subsequent experiments.

#### 3.4.2. Effect of EPS on Cholesterol Absorption in Caco-2 Cells

A hypercholesterolemic cell model was established in Caco-2 cells using cholesterol and sodium cholate. The inhibitory effect of EPS on cholesterol absorption was evaluated by measuring changes in TC content within the cells. As shown in Figure 5c, TC levels were significantly elevated in the model group compared with the control group (*p* < 0.01), confirming the successful induction of the hypercholesterolemic state. Treatment with EPS effectively counteracted the cholesterol micelle-induced increase in cellular TC in a dose-dependent manner. Significant reductions in TC were observed at EPS concentrations of 40 μg/mL and 160 μg/mL (*p* < 0.01), with the higher dose demonstrating a more pronounced inhibitory effect.

#### 3.4.3. Effect of EPS on PPARγ, LXRα, and NPC1L1 mRNA Levels in Caco-2 Cells

As summarized in Table 4, following EPS treatment, mRNA expression levels of the transcription factor PPARγ and its downstream target LXRα were significantly upregulated, whereas expression of the cholesterol transporter gene NPC1L1 was markedly downregulated. These in vitro results demonstrate that CPCM polysaccharides dose-dependently activate the PPARγ-LXRα signaling axis, consequently inhibiting the expression of the cholesterol transporter NPC1L1 and ultimately reducing cholesterol absorption in intestinal cells.

## 4. Discussion

The pathological feature of T2DM is the imbalance of glucose and lipid metabolism homeostasis, and its regulatory mechanism involves complex interactions among the intestinal flora, host cell signaling pathways, and immune–inflammatory responses [31]. The discovery of natural intervention agents with both hypoglycemic and lipid-lowering activities has become a research hotspot in the field of metabolic diseases. In this study, CPCM, a composite probiotic derived from fermented camel milk, was used as the research object. Through bidirectional validation using the in vivo *db/db* mice model and the in vitro Caco-2 cell model, combined with multi-omics technologies and molecular intervention approaches, this study systematically reveals the dual mechanisms by which CPCM regulates glucose and lipid metabolism in T2DM via the PPARγ-LXRα-NPC1L1 signaling pathway.

Our data showed that T2DM induces typical intestinal dysbiosis. More importantly, at both the genus and species levels, CPCM significantly promoted the colonization and proliferation of well-recognized beneficial bacteria, such as *Lactobacillus*, *Ligilactobacillus*, and *Akkermansia*, among which *Ligilactobacillus murinus* emerged as the dominant species after intervention. A large body of literature has reported that these bacterial genera are closely associated with improving intestinal barrier function, alleviating inflammation, and regulating glucose and lipid metabolism [32,33,34]. The function of this reshaped microbial system has also undergone beneficial transformations accordingly. Metagenomic analysis revealed the profound remodeling effect of CPCM on the metabolic functions of the intestinal flora. The results showed that CPCM significantly upregulated the acetyl-CoA transferase gene and enhanced the synthesis capacity of acetate and propionate. It should be explicitly noted that this conclusion is based on metagenomic gene abundance analysis and remains to be directly validated by targeted quantitative detection of SCFAs such as acetate, propionate, and butyrate in fecal or cecal contents. Future studies will use metabolomic techniques to quantitatively analyze SCFAs and the bile acid profile so as to further validate the inferences of this study and deeply elucidate the potential role of bile acid signaling in this context. More importantly, the changes in these microbial functional genes were significantly correlated with the expression of key proteins in the hepatic PPAR pathway. Metagenomic data also indicated that the abundance of beneficial bacteria such as *Akkermansia* was significantly increased after CPCM intervention. Regarding the controversial results of the *Bacillota*/*Bacteroidota* ratio at the phylum level (consistent with the study by Larsen et al. [35], but in contrast to the findings of Bahar-Tokman H et al. [36]), our interpretation is that the enrichment of beneficial bacteria mediated by CPCM may reconstruct the metabolic synergy structure among the microbiota. The metabolic status cannot be evaluated solely based on the single ratio previously used for microbiota assessment; changes in microbial functional genes may better reflect the regulatory effects after intervention than the community structure ratio.

In addition, we observed an interesting phenomenon: the abundance of the butyrate kinase gene in the T2DM model group was significantly higher than that in the control group. This result is consistent with the findings demonstrating metabolic homeostasis restoration. We speculate that CPCM does not simply enhance microbial functions but systematically improves the host metabolic environment, such as reducing systemic inflammation and improving insulin resistance, thereby alleviating metabolic pressure on the intestinal flora so that it no longer needs to perform compensatory high-load work. This finding is consistent with the observations of He et al. in a metabolic disorder model, which may reflect a compensatory response of the gut microbiota to counteract the imbalance of host energy homeostasis [37]. As a key energy source for the colon, the upregulation of the butyrate synthesis pathway represents a vital link in microbiota–host interactions [38]. Following CPCM intervention, the abundance of this gene reverted to normal levels, accompanied by the upregulation of acetyl-CoA transferase activity. This indicates that the effect of CPCM is systemic remodeling rather than a single stimulatory action. Consequently, CPCM intervention led to an overall improvement in the short-chain fatty acid profile, and acetate, propionate, and butyrate exert a synergistic effect in regulating immunity, intestinal barrier function, and metabolism [39]. Therefore, this functional shift from a “compensatory” state to a “homeostatic” state may represent one of the core mechanisms underlying the efficacy of CPCM. Combined with hepatic proteomic analysis, the results of our study found that T2DM modeling led to the dysregulation of the hepatic PPAR signaling pathway, in which key proteins such as *ACOX1/2*, *FABPs*, and retinoid X receptor α (*RXRα*) were downregulated. These changes in related proteins are associated with abnormal hepatic lipid metabolism. However, CPCM intervention does not simply activate this pathway but restores the homeostasis of the pathway, that is, reverses the abnormally expressed protein levels to the normal state. The results of the Kyoto Encyclopedia of Genes and Genomes (KEGG) pathway enrichment analysis showed that CPCM can reconstruct hepatic metabolic homeostasis through the PPAR signaling pathway, which further confirms the role of CPCM in stabilizing metabolism.

The causes of this series of hepatic changes have been attributed to the intestines. In vivo experiments have confirmed the gut–liver-mediated mechanism of action, but can CPCM itself also directly act on the host? We answered this question through in vitro experiments. We confirmed that the polysaccharide component of CPCM can directly inhibit cholesterol absorption in Caco-2 cells, and this effect is mediated by activating PPARγ-LXRα and subsequently downregulating NPC1L1 expression. Quantitative polymerase chain reaction (qPCR) results showed that polysaccharide treatment significantly upregulated the mRNA expression of PPARγ and its downstream target LXRα in Caco-2 cells in a dose-dependent manner and significantly downregulated the gene expression of NPC1L1, a key rate-limiting transporter for cholesterol absorption. Our research team previously examined the expression of related proteins in the liver of *db/db* mice using Western blotting. The results showed that CPCM significantly upregulated the expression of PPARγ and LXRα and downregulated the expression of NPC1L1 in the colon [24], which is consistent with the findings of the present study. These changes in gene expression clearly demonstrate the pathway of “PPARγ ↑ → LXRα ↑ → NPC1L1 ↓” at the molecular level, providing a reasonable mechanistic explanation for the inhibition of cholesterol absorption by CPCM.

In the present study, CPCM intervention reversed the dysregulated expression of key proteins in the hepatic PPAR signaling pathway and activated the PPARγ-LXRα-NPC1L1 signaling axis in intestinal epithelial cells, thereby inhibiting cholesterol absorption in T2DM mice. This finding is consistent with the research by Huang and Zheng, who demonstrated that the probiotic *Lactobacillus* acidophilus could downregulate NPC1L1 expression and reduce intestinal cholesterol uptake through an LXR-dependent pathway [40]. However, unlike the reports in some studies that LXR activation is accompanied by elevated HDL-C levels [41,42], no significant increase in HDL-C was observed in our study.

We attribute this observation to the following reasons: First, the effects of the LXR signaling pathway exhibit tissue specificity and dependence on disease status. In intestinal epithelial cells, LXR activation primarily promotes cholesterol efflux by inducing the expression of *ABCG5/G8* and reduces cholesterol absorption by downregulating NPC1L1 expression, with its direct effects manifested in local cholesterol transport rather than systemic HDL metabolism [32]. This study focused on the regulation of intestinal epithelial cells and the gut–liver axis, and the promotive effect of LXR on hepatic HDL-C synthesis may have been partially offset or delayed by pathological conditions such as hepatic lipid metabolism disorder and insulin resistance in the T2DM state [43]. Second, relevant studies have indicated that in diabetic or high-fat diet animal models, although LXR can be activated, the response of some of its downstream target genes (e.g., genes involved in HDL synthesis) may be impaired, leading to insignificant changes in HDL-C levels, whereas the regulation of genes associated with cholesterol absorption and excretion remains relatively intact [44]. In this study, CPCM may preferentially regulate cholesterol absorption via the intestinal LXR-NPC1L1 axis—a pathway that retains functional activity in the T2DM state—whereas the hepatic HDL metabolic pathway may require a longer duration or more potent intervention to achieve a significant improvement.

Based on all the above findings, we can reveal the integrated mechanism by which CPCM improves glucose and lipid metabolism in T2DM: First, after CPCM enters the intestine, its bacterial components reshape the flora structure through ecological effects such as competition and symbiosis, restoring and enhancing the SCFA synthesis function of the flora (butyrate is reversed to normal levels, and acetate/propionate are upregulated). These metabolites indirectly restore the function of the hepatic PPAR pathway through the gut–liver axis. At the same time, the active polysaccharides of CPCM directly act on intestinal epithelial cells, activate the intracellular PPARγ-LXRα signal, downregulate NPC1L1, and directly inhibit cholesterol absorption. Ultimately, these direct and indirect signals converge in the liver and synergize with the anti-inflammatory and thermogenic effects induced by high doses, as well as the endocrine regulatory effects induced by low doses, to comprehensively improve glucose and lipid metabolism.

This study has established a research system of “in vivo flora remodeling–in vitro cell validaton”, which not only reveals the indirect mechanism by which CPCM indirectly regulates host pathways through microbial metabolites but also clarifies its direct mechanism of action on intestinal epithelial cells, thus defining the regulatory mechanism. Meanwhile, this study also has certain limitations: firstly, the in vitro experiments failed to simulate the complex microenvironment of the interaction between intestinal flora and intestinal epithelial cells in vivo, and thus could not comprehensively evaluate the synergistic enhancement effect of microbial metabolites (such as SCFAs and secondary bile acids) on the regulatory effects of CPCM; secondly, NPC1L1 protein is significantly expressed in the human liver and is involved in the reabsorption of biliary cholesterol, whereas its expression level is extremely low in the liver of *db/db* mice, which makes the improvement of hepatic lipid metabolism observed in mice in this study mainly attributed to the indirect effect of CPCM inhibiting intestinal NPC1L1-mediated cholesterol absorption rather than direct regulation on the liver; although human Caco-2 cell experiments have confirmed that CPCM can downregulate intestinal NPC1L1 expression and inhibit cholesterol uptake, the potential regulatory effect of CPCM on hepatic biliary cholesterol reabsorption still needs to be further verified in more clinically relevant models; finally, metagenomic and proteomic data are typical high-dimensional omics data, and no unified and mature method is currently available for a priori statistical power calculation in the academic community. Therefore, a priori power analysis was not performed in this study, which warrants further validation and optimization with more robust statistical strategies and experimental designs in the future. The core findings confirm that the effects of CPCM exhibit distinct dose dependence and multi-pathway synergy characteristics. In vivo, CPCM restores the function of the hepatic PPAR signaling pathway impaired by diabetes through the “gut–liver axis” by reshaping the intestinal flora structure (e.g., enriching beneficial bacteria such as *Lactobacillus* and *Akkermansia*) and their functions (regulating SCFA synthesis genes), as evidenced by the successful reversal of the expression of key proteins such as ACOX and FABPs. In vitro, we further revealed its direct mechanism of action: the active polysaccharide components of CPCM can directly activate the PPARγ-LXRα-NPC1L1 signaling pathway in intestinal epithelial cells, independent of the flora. In conclusion, through the integration of animal models, multi-omics technologies, and in vitro experiments, this study clarifies the integrated mechanism by which CPCM improves glucose and lipid metabolism in T2DM mice.

## 5. Conclusions

In summary, this study confirms that CPCM ameliorates lipid metabolic dysfunction in *db/db* mice via a dual regulatory mechanism. CPCM reshapes the gut microbiota by enriching beneficial taxa (*Lactobacillus*, *Akkermansia*) and reducing harmful bacteria (*Clostridium*), restoring the metabolic homeostasis of the flora and alleviating intestinal low-grade inflammation. Concomitantly, CPCM normalizes the expression of key proteins in the hepatic PPAR signaling pathway, and its active component, extracellular polysaccharides, directly activates the PPARγ-LXRα-NPC1L1 axis in intestinal epithelial cells to inhibit cholesterol absorption. These in vivo and in vitro findings collectively elucidate that CPCM modulates lipid metabolism through gut microbiota-mediated indirect regulation and direct targeting of intestinal epithelial cell signaling, with high-dose CPCM exerting more pronounced effects on microbiota remodeling and anti-inflammation. This work provides a novel mechanistic basis for probiotic-based interventions in T2DM-associated lipid metabolic disorders.

## Figures and Tables

**Table 1:** Impact of CPCM on fasting blood glucose levels (FBG) in *db/db* mice.

Group	Before Treatment (mmol/L)	Week 2 (mmol/L)	Week 4 (mmol/L)	Week 6 (mmol/L)	Week 8 (mmol/L)
Control	5.36 ± 1.15	6.49 ± 1.11	6.20 ± 1.04	6.28 ± 0.53	6.70 ± 0.83
Model	17.50 ± 2.10 ***	24.96 ± 2.25 ***	27.43 ± 2.01 ***	27.33 ± 1.68 ***	26.41 ± 1.88 ***
Metformin	17.31 ± 1.48	24.70 ± 2.00	25.01 ± 2.44 ^#^	24.19 ± 1.65 ^###^	20.96 ± 1.84 ^###^
Low dose	17.41 ± 1.51	24.88 ± 2.18	26.78 ± 1.63	25.08 ± 1.46 ^##^	24.29 ± 2.03 ^#^
High dose	17.78 ± 1.46	24.21 ± 2.20	26.51 ± 1.68	25.70 ± 1.18 ^#^	23.78 ± 1.91 ^##^

Data are presented as mean ± standard deviation (SD) (n = 8). *** *p* < 0.001 vs. control group; ^#^ *p* < 0.05, ^##^ *p* < 0.01, and ^###^ *p* < 0.001 vs. model group. These statistical comparisons highlight the significant differences between the groups in the study.

**Table 2:** Impact of CPCM on oral glucose tolerance test (OGTT) and area under the curve (AUC) in *db/db* mice.

Group	0 min (mmol/L)	30 min (mmol/L)	60 min (mmol/L)	90 min (mmol/L)	120 min (mmol/L)	AUC
Control	6.28 ± 0.70	12.60 ± 1.26	9.80 ± 1.32	8.39 ± 1.07	6.59 ± 1.17	1116.88 ± 61.09
Model	27.78 ± 2.09 ***	33.50 ± 2.16 ***	32.25 ± 2.53 ***	29.53 ± 2.13 ***	27.34 ± 1.78 ***	3685.38 ± 89.06 ***
Metformin	20.36 ± 3.11 ^###^	28.65 ± 1.36 ^###^	26.74 ± 1.07 ^##^	24.69 ± 1.08 ^###^	21.26 ± 1.18 ^###^	3026.88 ± 119.90 ^###^
Low dose	21.60 ± 2.17 ^###^	30.46 ± 1.76 ^##^	28.45 ± 1.23 ^##^	26.10 ± 1.30 ^###^	23.41 ± 1.27 ^###^	3225.88 ± 153.29 ^###^
High dose	21.95 ± 1.02 ^###^	29.83 ± 2.56 ^###^	27.00 ± 1.64 ^##^	25.64 ± 1.91 ^###^	22.19 ± 2.12 ^###^	3136.13 ± 166.92 ^###^

Data are presented as mean ± standard deviation (SD) (n = 8). *** *p* < 0.001 vs. control group; ^##^ *p* < 0.01, ^###^ *p* < 0.001 vs. model group. These statistical comparisons highlight the significant differences between the groups in the study.

**Table 3:** Impact of T2DM on the expression of key proteins in the liver PPAR signaling pathway.

Symbol	Protein Name	Kegg Genes	Regulate	*p*-Value
*Acox1*	Peroxisomal acyl-coenzyme A oxidase 1	mmu:11430	down	<0.05
*Acox2*	Peroxisomal acyl-coenzyme A oxidase 2	mmu:93732	down	<0.01
*Fabp1*	Fatty acid-binding protein, liver	mmu:14080	down	0.069
*Fabp2*	Fatty acid-binding protein, intestinal	mmu:14079	down	<0.01
*Fabp4*	Fatty acid-binding protein, adipocyte	mmu:11770	down	<0.01
*Fabp7*	Fatty acid binding protein 7, brain	mmu:12140	down	0.053
*Rxra*	Retinoic acid receptor RXR	mmu:20181	down	<0.01

**Table 4:** Impact of EPSs on PPARγ, LXRα, and NPC1L1 mRNA levels in Caco-2 cells.

Group	PPARγ	LXRα	NPC1L1
Control	1.0	1.0	1.0
10 μg/mL	1.3 *	1.1	0.9
40 μg/mL	1.7 **	1.9 **	0.5 *
160 μg/mL	2.9 **	2.5 **	0.4 *

* *p* < 0.05, ** *p* < 0.01. All data in the table are from three independent experiments (n = 3).

## Data Availability

Data are contained within the article. Further inquiries can be directed to the corresponding author.

## Supplementary Materials

The following supporting information can be downloaded at: https://www.mdpi.com/article/10.3390/metabo16030157/s1, Table S1: The primer sequences of the target genes; Table S2: Impact of CPCM on body weight in *db/db* mice; Table S3: The relative abundance of identified microbial taxa; Table S4: The top ten differentially expressed proteins (DEP); Table S5: Strain identification and deposit information of the CPCM consortium.

## Abbreviations

The following abbreviations are used in this manuscript:

AUC	Area under the curve
ANOVA	Analysis of variance
BSA	Bovine Serum Albumin
BH	Benjamini–Hochberg
CPCM	Composite probiotics derived from fermented camel milk
CDCA	Chenodeoxycholic acid
CP	C-peptide
Chao index	Chao diversity index
DM	Diabetes mellitus
DEP	Differentially expressed proteins
DMSO	Dimethyl sulfoxide
DMEM	Dulbecco’s Modified Eagle Medium
EPS	Exopolysaccharides
ELISA	Enzyme-linked immunosorbent assay
FXR	Farnesoid X receptor
FABP	Fatty acid-binding protein
FBG	Fasting blood glucose
GLP-1	Glucagon-like peptide-1
GPR43/41	G protein-coupled receptor
HbA1c	Glycated Hemoglobin A1c
HDL-C	High-density lipoprotein cholesterol
IDF	International Diabetes Federation
IACUC	Institutional Animal Care and Use Committee
KEGG	Kyoto Encyclopedia of Genes and Genomes
LXRα	Liver X receptor α
LPS	Lipopolysaccharide
LCA	Lithocholic acid
LAB	Lactic acid bacteria
LDL-C	Low-density lipoprotein cholesterol
LC–MS	Liquid chromatography-mass spectrometry
MyD88	Myeloid differentiation factor 88
MTT	3-(4,5-dimethylthiazol-2-yl)-2,5-diphenyltetrazolium bromide
NPC1L1	Niemann–Pick C1-like 1
NF-κB	Nuclear factor kappa-B
NMDS	Non-metric multidimensional scaling
PPARγ	Peroxisome proliferator-activated receptor γ
PYY	Peptide tyrosine tyrosine
PCoA	Principal coordinate analysis
OGTT	Oral glucose tolerance test
PBS	Phosphate-buffered saline
qPCR	Quantitative real-time PCR
RXRα	Retinoid X receptor α
SCFA	Short-chain fatty acid
SPF	Specific pathogen-free
SD	standard deviation
T2DM	Type 2 diabetes mellitus
TGR5	Takeda G Protein-Coupled Receptor 5
TLR	Toll-like receptor
T-α-MCA	Tauro-α-muricholic acid
T-β-MCA	Tauro-β-muricholic acid
TC	Total cholesterol
TG	Triglycerides
TFA	Trifluoroacetic acid
